# Case report: BA.1 subvariant showing a BA.2-like pattern using a variant-specific PCR assay due to a single point mutation downstream the spike 69/70 deletion

**DOI:** 10.1186/s12985-022-01883-2

**Published:** 2022-10-27

**Authors:** Carlos Daviña-Nuñez, Sonia Pérez-Castro, Lucía Martínez-Lamas, Jorge Julio Cabrera-Alvargonzález, Sonia Rey-Cao, Raquel Carballo-Fernandez, Montse Godoy-Diz, Leticia López-Bóveda, Victor del Campo-Pérez, Silvia Suárez-Luque, Benito Regueiro-García

**Affiliations:** 1 Universidade de Vigo. Microbiology and Infectology Research Group, Galicia Sur Health Research Institute (IIS Galicia Sur), Vigo, Spain; 2 Microbiology and Infectology Research Group, Galicia Sur Health Research Institute (IIS Galicia Sur). Microbiology Department, Complexo Hospitalario Universitario de Vigo (CHUVI), SERGAS, Vigo, Spain; 3 Microbiology Department, Complexo Hospitalario Universitario de Vigo (CHUVI), SERGAS, Vigo, Spain; 4 Rheumatology and Immune-mediated Diseases Research Group, Galicia Sur Health Research Institute (IIS Galicia Sur). Preventive Medicine Department, Complexo Hospitalario Universitario de Vigo (CHUVI), SERGAS, Vigo, Spain; 5 Dirección Xeral de Saúde Pública, Xunta de Galicia, Consellería de Sanidade, Santiago de Compostela, A Coruña Spain; 6 Microbiology and Infectology Research Group, Galicia Sur Health Research Institute (IIS Galicia Sur), Vigo, Spain

**Keywords:** SARS-CoV-2, Omicron, Next generation sequencing, Variant-specific PCR, Surveillance, BA.1, BA.4, BA.5

## Abstract

**Background:**

SARS-CoV-2 variant tracking is key to the genomic surveillance of the COVID-19 pandemic. While next-generation sequencing (NGS) is commonly used for variant determination, it is expensive and time-consuming. Variant-specific PCR (vsPCR) is a faster, cheaper method that detects specific mutations that are considered variant-defining. These tests usually rely on specific amplification when a mutation is present or a specific melting temperature peak after amplification.

**Case presentation:**

A discrepant result between vsPCR and NGS was found in seventeen SARS-CoV-2 samples from Galicia, Spain. A cluster of BA.1 Omicron SARS-CoV-2 variant showed a BA.2-like melting temperature pattern due to a point mutation (C21772T) downstream the deletion of the spike amino acids 69/70. As the 69/70 deletion is widely used for differentiation between BA.1 and BA.2 by vsPCR, C21772T can cause BA.1 samples to be misinterpreted as BA.2. Over a thousand BA.1 sequences in the EpiCoV database contain this mutation.

**Conclusions:**

To our knowledge, this is the first case of a point mutation causing a vsPCR algorithm to misclassify BA.1 samples as BA.2. This is an example of how mutations in the probe target area of vsPCR tests based on melting curve analysis can lead to variant misclassification. NGS confirmation of vsPCR results is relevant for the accuracy of the epidemiological surveillance. In order to overcome the possible impact of novel mutations, diagnostic tools must be constantly updated.

**Supplementary Information:**

The online version contains supplementary material available at 10.1186/s12985-022-01883-2.

## Background

During the severe acute respiratory syndrome coronavirus 2 (SARS-CoV-2) pandemic, epidemiological surveillance teams have relied on next-generation sequencing (NGS) to track the spread of new variants and mutations. As a cheaper, faster alternative, commercial providers have developed assays in order to detect mutations that define a specific variant (variant-specific PCR, vsPCR) [1, 2]. These methods rely on melting curve assays or multiplex quantitative RT-PCRs. Spike mutations commonly targeted in these assays include E484K (specific of VOCs Beta and Gamma), N501Y (specific of VOCs Alpha, Beta, Gamma and Omicron) or L452R (specific of VOCs Delta and Omicron BA.4/5). One noteworthy example is the case of the deletion of nucleotides 21,765–21,770, causing the deletion of spike amino acids 69 and 70 (69/70del). First detected in Alpha variant, it causes a false-negative result in the S-gene of some RT-PCR detection kits (S-Gene Target Failure, SGTF). Detection of the 69/70del became relevant again as it appears in Omicron subvariant BA.1 but not in BA.2 (PANGO lineage), therefore being a perfect candidate for the determination of Omicron subvariant using a vsPCR assay [3].

Here, we report a cluster of seventeen SARS-CoV-2 strains assigned by next generation sequencing as BA.1 that were erroneously designated by one vsPCR assay as BA.2 due to a detection failure of the 69/70 deletion. Our objective was to look for point mutations in the region surrounding the deletion that could explain this discrepancy. This is a new example of how point mutations can alter variant determination results via vsPCR, and of the potential implications for vsPCR-based SARS-CoV-2 surveillance.

## Case presentation

In March 2022, 17 patients diagnosed with SARS CoV-2 infection at the Complexo Hospitalario Universitario de Vigo (Vigo, Galicia, Spain) showed discrepant SARS-CoV-2 variant results between an automated vsPCR assay and NGS. For the vsPCR assay, RNA extraction was performed with GXT 96 × 3 Extraction kit v1.0 (Hain Lifescience GmbH, Nehren, Germany) on the GenoXtract ® fleXT instrument (Bruker) and PCR was performed with Fluorotype SARS-CoV-2 varID Q (Hain Lifescience GmbH, Nehren, Germany) on the FluoroCycler XT (Hain Lifescience) instrument according to manufacturer’s instructions. The mutation A67V (C21762T) upstream the 69/70del is usually present in BA.1 variants. In consequence, tests like the Hain test used for this study updated their reagents to detect the specific combination of A67V + 69/70del [4, 5]. A false-negative result in the A67V 69/70del-specific PCR was obtained and the presence of the BA.2 variant was determined by the interpretation algorithm.

For NGS, RNA extraction from the same samples was performed using QIASymphony DSP Virus Pathogen Midi kit (Qiagen, Hilden, Germany) according to manufacturer’s instructions. Whole-Genome Sequencing was performed using the COVIDSeq Assay Kit (Illumina Inc., San Diego, CA, USA). Sixteen samples were sequenced by MiSeq using MiSeq Reagents v3 (Illumina Inc.) and a Hamilton instrument for automated library preparation, while one sample was sequenced on an iSeq 100 using iSeq 100 I1 Reagents (Illumina Inc.) after manual library preparation. Bioinformatic analysis was performed with a custom made pipeline and UShER genome was used for variant determination (Supplementary data 1). Upon NGS analysis, the samples were characterised as Omicron BA.1.1.14 with the synonymous mutation C21772T immediately downstream the 69/70del. We suggest that this mutation prevented the detection of the 69/70del. Deletion 69/70 leads to the loss of the amino acids histidine (H) and valine (V). Given that ATA, ATC and ATT all translate into isoleucine (I), mutation C21772T did not cause a substitution in the amino acid sequence (Table 1).

**Table 1:** Nucleotide changes associated with the 69/70 codon deletion of spike of SARS CoV-2 for BA.1 and BA.2 variants and for BA.1 variant with C21772T mutation

Variant	Nucleotide sequence (21,761–21,775) (codons 67–71)	Amino acid sequence (spike 67–71)
**Wuhan-Hu-1**	GCTATACATGTCTCT	AIHVS
**BA.1**	G**T**TA‒‒‒‒‒‒TCTCT	VI‒‒S
**BA.1 + C21772T**	G**T**TA‒‒‒‒‒‒T**T**TCT	VI‒‒S
**BA.2**	GCTATACATGTCTCT	AIHVS

Mutation C21762T (characteristic of BA.1 subvariant) and mutation C21772T (described in this paper) are highlighted

The discrepant samples belonged to a cluster of BA.1.1.14 with G9049A which accounts for 96% of the BA.1.1.14 samples in Spain [6]. We identified 19 samples in the cluster presenting mutation C21772T, all of them detected in Galicia, Spain. Seventeen out of 19 samples were detected in our laboratory. A multiple sequence alignment was performed to confirm that the samples were monophyletic (Supplementary Fig. 1). The multiple sequence alignments and phylogenetic trees were performed and constructed on R (version 4.1.1, https://cran.r-project.org/) using the packages ggtree [7], ggplot [8], msa [9], and custom-made code.

Contact tracing showed that 10 sequences pertained to high school students, and 4 of them were epidemiologically related. All GISAID accession IDs for these 17 sequences are listed in Supplementary Table 1. It is noteworthy that, upon alignment against the Wuhan-Hu-1 reference, some alignments misplace the 69/70del, and thus, the mutation is numbered as A21766T instead of C21772T (Supplementary Table 2). That is the case of databases such as CoVSpectrum and Nextclade [6, 10]

**Fig. 1:**
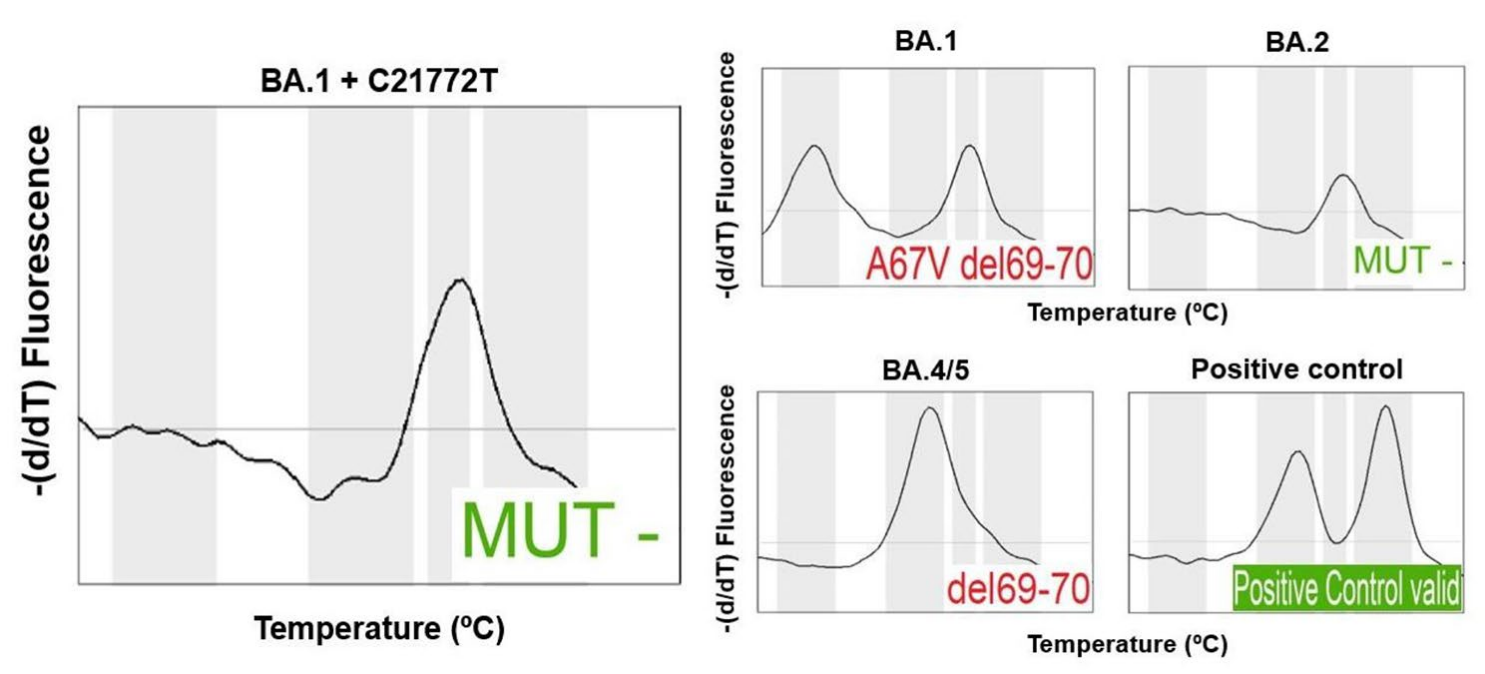
**BA.2-like result of the cluster BA.1 + C21772T in the Fluorotype SARS-CoV-2 varIDQ (Hain Lifescience). Left**: Melting curve for a representative sample of the cluster BA.1 + C21772T. **Right**: Melting curves for BA.1, BA.2, BA.4/5 and positive control samples. For all cases, the result given by the interpretation algorithm is shown on the bottom right of each melting curve plot. BA.1 gives a positive result in A67V + 69/70del, while BA.4/5 gives a positive result in 69/70del, as expected. The mutation C21772T in the BA.1 subvariant leads to the removal of the melting peak and a pattern similar to the BA.2 subvariant. The algorithm interprets the result of the BA.1 + C21772T samples as negative for both A67V-69/70del and 69/70del

**Fig. 2:**
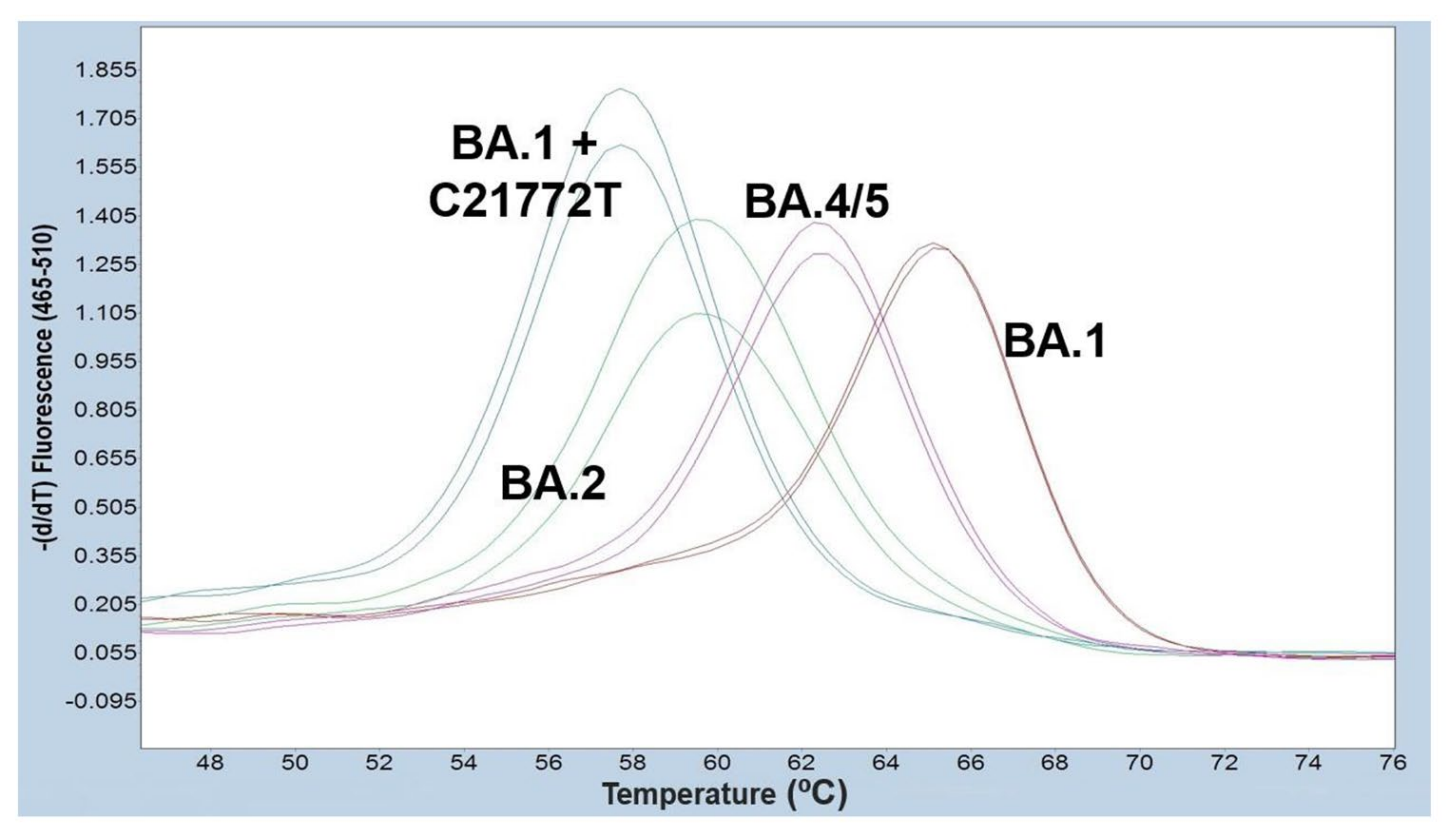
**Non-interpretable result of the cluster BA.1 + C21772T in the VirSNiP SARS-CoV-2 A67V 69/70del (TibMolBiol) assay.** Melting curves for BA.1, BA.2 and BA.4/5 correspond to the expected peaks for 67 V-69/70del, wild-type, and 69/70del, respectively. BA.1 + C21772T presents a temperature peak shift from BA.1 of 7.3 ºC yielding a non-interpretable result in the A67V-69/70del detection

In order to confirm the discrepancy between NGS and vsPCR, the 17 BA.1 + C21772T samples were re-tested in the Hain assay. In this second test, the QIAsymphony nucleic acid extract obtained for NGS was used, in order to avoid any discrepancies caused by the different extraction methods. The second extract gave the same false-negative result and BA.2 interpretation (Fig. 1, Supplementary Table 3). To support this result, we tested the samples with a second vsPCR assay. A non-interpretable result due to a shift in melting temperature was observed with VirSNiP SARS-CoV-2 A67V 69/70del and LightCycler 1-step RT-polymerase (TibMolBiol, Berlin, Germany) on the cobas z480 instrument (Roche Diagnostics, Basel, Switzerland) (Fig. 2, Supplementary Table 4)

## Discussion and conclusions

The spike deletion 69/70 has been used for Omicron detection since its emergence because it was specific for BA.1 subvariant and a differential characteristic from the Delta variant circulating at that time. Afterwards, it was also useful to differentiate between BA.1 (69/70del positive) and BA.2 (69/70del negative) subvariants. Some commercial tests rely on the false-negative result on S-gene to detect BA.1 (SGTF) [3], whereas others such as the ones used in this paper offer a specific RT-PCR approach to detect the A67V-69/70del. In the same manner that the 69/70del causes SGTF, new mutations can cause a failure in specific vsPCRs.

Commonly, RT-PCR tests for detection of SARS-CoV-2 are based on fluorescent probes binding to a specific amplicon, although some assays have been developed using dsDNA binding dyes such as SYBR Green [11, 12]. Examples of mutations and deletions in both primer and probe target sequence affecting RT-PCR and melting temperature results have been previously reported.

Amongst some of these reports, a pre-Omicron case report showed that a single point mutation in the probe binding sequence can interfere with SARS-CoV-2 detection on a RT-PCR diagnostic test [13]. Other cases of non-amplification in diagnostic assays include the deletion 69/70 causing non-amplification of the Spike gene in some tests (SGTF) [3], or the Hain Lifesciences test used in this paper, that shows a false-negative result for N-gene amplification on the Omicron variant due to deletion 31–33 in N gene overlapping with the primer sequence [5].

Single nucleotide polymorphisms (SNPs) affecting test results have also been shown for melting curve-based assays. For instance, as presented in this publication, mutation C21762T of BA.1, overlapping with the probe sequence in both Hain and VirSNiP [14, 15], causes a shift in melting temperature for detection of 69/70del. Additionally, in a recent publication 19 laboratories tested an Alpha sample with mutation S:G75V for 69/70del with different unspecified assays. Five of them gave an unclear result, while three of them presented an incorrect result (false-negative), showing the impact of a single mutation in vsPCR interpretation [16]. Amongst other examples, a shift in melting temperature of 5 ºC for E484K and 8 ºC for E484A compared to wild-type has been shown using a VirSNiP assay [17]. Mutation N501Y for Spike in Omicron is reported as false-negative using both the VirSNiP and the Hain LifeSciences vsPCR, likely due to additional mutations surrounding amino acid 501 [17, 18]. According to information provided by the manufacturers, in the case of the Hain assay, mutation Q489R is responsible for the false-negative result [14].

Regarding the case studied in this publication, the deletion 69/70 and the surrounding region is targeted by the fluorescent probe and not the primers, according to the instructions manual of both tests [14, 15] and information provided by the manufacturers. We observed that the mutation C21772T, two bases downstream from the deletion, is the common SNP for all samples with the melting peak phenomena described here. This shows one more example of how a point mutation can alter the result of a vsPCR and, in this specific case, how it can cause the misclassification of a SARS-CoV-2 variant.

As of May 16th, there are 1315 sequences from BA.1 with the 69/70del and C21772T mutation, belonging to different BA.1 subvariants [6] suggesting that this mutation could have spontaneously appeared at different times. If over a thousand BA.1 variants with the mutation C21772T have been detected since the appearance of the Omicron variant, it could be possible that there was an underestimation of the frequency of BA.1 in surveillance services that relied mainly on melting curve-based vsPCR assays for variant tracking. In addition, while this paper is focused on commercial tests used for genomic surveillance of SARS-CoV-2, the same issue can arise for in-house methods developed for wastewater samples [19]. This is, to the best of our knowledge, the first report of C21772T causing a false-negative result in 69/70del-targeted RT-PCR assays. It is highly likely that other mutations can cause similar effects in SARS-CoV-2 variant tracking in the future.

Our example is only one of many challenges arising for variant tracking with PCR-based subvariant surveillance for the Omicron wave of SARS-CoV-2: as total cases increase, so do the chances of diagnostic PCR tests affected by a novel mutation such as the ones mentioned above. Also, the recently discovered BA.4 and BA.5 Omicron subvariants show a new mutational pattern not expected by the interpretation software, making variant determination with vsPCR increasingly complex. For example, mutation A67V makes possible the differentiation between BA.1 and BA.4/5, as explained in this report. However, BA.4 and BA.5 are genetically identical in the area surrounding 69/70del, so other targets are needed for lineage determination. Finally, recombinant SARS-CoV-2 variants such as PANGO lineage XD or XE, which combine AY.4/BA.1 and BA.1/BA.2 respectively, increase the complexity for taxonomic classification of the virus using methods based in the detection of combinations of single point mutations.

As Omicron subvariants show a high number of spike mutations, and given all the complexities mentioned, we recommend commercial kit providers to increase their available targets in order to obtain a more specific differentiation between SARS-CoV-2 lineages and sublineages with vsPCR assays. Increasing the number of targets in a single reaction is challenging, given that too many fluorophores would lead to overlapping emission spectra and suboptimal results. While this has been solved by making two reactions per sample by some manufacturers, it can be more costly and time-consuming. Another commercial approach is to offer a wide range of targets and fluorophores in order to be prepared for the detection of any new variant that arises. A constant update of the available targets and periodic checks of their performance with circulating SARS-CoV-2 isolates are key to maintaining optimal epidemiological surveillance.

The speed in the detection of new mutations and variants is crucial in order to prepare an adequate response to the new circumstances of the COVID-19 pandemic. For instance, the appearance of variants Delta and Omicron caused a change in strategies as these variants were responsible for an increased number of cases and hospitalisations due to increased transmissibility and immune escape [20–23]. In the current phase of the pandemic, where testing numbers have been decreasing [24], the odds of missing relevant novel mutations are higher, as the rate of undetected and unsequenced viral genomes increases. It is therefore essential to keep adequate surveillance with the resources available. As mutations in the target area of vsPCR tests based on melting curve analysis can lead to variant misclassification, NGS confirmation of PCR results is still relevant for the accuracy of the epidemiological surveillance.

## Electronic supplementary material

Below is the link to the electronic supplementary material.

Supplementary Material 1

## Data Availability

The datasets used and/or analysed during the current study are available from the corresponding author on reasonable request. The genetic sequences from the samples analysed are available on GISAID and all ID accession numbers can be found in Supplementary Table 1.

## Abbreviations

NGS: Next-Generation Sequencing
vsPCR: variant-specific PCR
SARS-CoV-2: Severe Acute Respiratory Syndrome CoronaVirus 2
VOCs: Variants of Concern
69/70del: spike gene amino acid 69–70 deletion
SGTF: Spike Gene Target failure
SNP: Single Nucleotide Polymorphism
