# Supplementary material for: Case report: BA.1 subvariant showing a BA.2-like pattern using a variant-specific PCR assay due to a single point mutation downstream the spike 69/70 deletion
Source: Virol J. 2022 Oct 27;19:168. doi: 10.1186/s12985-022-01883-2 (PMC9610356; doi:10.1186/s12985-022-01883-2)
Supplement: Supplementary file 1 — Supplementary Material 1 [file 12985_2022_1883_MOESM1_ESM.docx]

**Supplementary table 1: GISAID accession numbers to all 17 samples with C21772T sequenced in Vigo, Spain.** All samples in the list have been uploaded by our facility.

| **GISAID accession ID** |
| --- |
| EPI_ISL_11247810 |
| EPI_ISL_11247815 |
| EPI_ISL_11220353 |
| EPI_ISL_11247854 |
| EPI_ISL_11247888 |
| EPI_ISL_12057144 |
| EPI_ISL_12130350 |
| EPI_ISL_11247973 |
| EPI_ISL_11248021 |
| EPI_ISL_10967458 |
| EPI_ISL_10927108 |
| EPI_ISL_11247980 |
| EPI_ISL_10927129 |
| EPI_ISL_12130307 |
| EPI_ISL_10672192 |
| EPI_ISL_10672181 |
| EPI_ISL_10252558 |

**Supplementary table 2:** **Misalignment of BA.1 + C21772T against reference Wuhan-Hu-1 causes databases to register C21772T as A21776T.** While both alignments are possible, the sequence with A21776T causes a displacement of 69/70del compared to the parental lineage BA.1 (from 21765-21760 to 21767-21762). Mutation C21772T (or A21776T) is highlighted.

| **Variant** | **Nucleotide sequence (21761-21776)** |
| --- | --- |
| **Wuhan-Hu-1** | GCTATACATGTCTCT |
| **BA.1** | GTTA‒‒‒‒‒‒TCTCT |
| **BA.1 + C21772T** | GTTA‒‒‒‒‒‒T**T**TCT |
| **BA.1 + A21766T (misalignment)** | GTTAT**T**‒‒‒‒‒‒TCT |

**Supplementary table 3. Interpretation algorithm for Omicron variants identification by variant specific PCR Hain’s Fluorotype SARS-CoV-2 varID Q.** Summary of SARS-CoV-2 spike amino acids pattern for the identification of Omicron variants using Hain’s Fluorotype SARS-CoV-2 varID Q ver 1.0 (Hain Lifescience GmbH, Nehren, Germany). RdRp is used as internal control of the PCR. This reagent is not able to detect the N501Y change in the case of the Omicron variants.

|  | **Fluorotype SARS-CoV-2 varID Q** | | | | | | |
| --- | --- | --- | --- | --- | --- | --- | --- |
|  | **RdRp** | **N** | **A67V 69/70del** | **69/70del** | **N501Y** | **E484K** | **D80A** |
| **BA.1** | **+** | **-** | **+** | **-** | **-** | **-** | **-** |
| **BA.1 + C21772T** | **+** | **-** | **-** | **-** | **-** | **-** | **-** |
| **BA.2** | **+** | **-** | **-** | **-** | **-** | **-** | **-** |

**Supplementary table 4: Average melting temperature peaks detected for Omicron variants using variant specific PCR VirSnip SARS-CoV-267V del69/70.** All peaks correlate with their expected position according to the genome of the variant (BA.1 - 67V 69/70del, BA.2 - no deletion, BA.4/5 - 69/70del). C21772T causes an average 7.3 ºC shift in melting temperature compared with parental BA.1.

| **​** | **VirSnip assay A67V 69/70del​** | |
| --- | --- | --- |
| ​ | **Melting temperature**  **peak expected**​ | **Melting temperature**  **peak detected**​ |
| **BA.1​** | 65.0-66.0 ºC​ | 65.2 ºC​ |
| **BA.1 + ​**  **C21772T​** | NA​ | 57.9 ºC​ |
| **BA.2​** | 60.0-61.0 ºC​ | 60.0 ºC​ |
| **BA.4/5​** | 62.5-63.5 ºC​ | 62.4 ºC​ |

**Supplementary Figure 1:** **Phylogenetic tree and sequences (nucleotides 21760-21780).** Subset of 84 BA.1.1.14 samples detected in Vigo, Galicia, Spain collected during March of 2022. BA.2 and Wuhan-Hu-1 variant are shown as outliers.


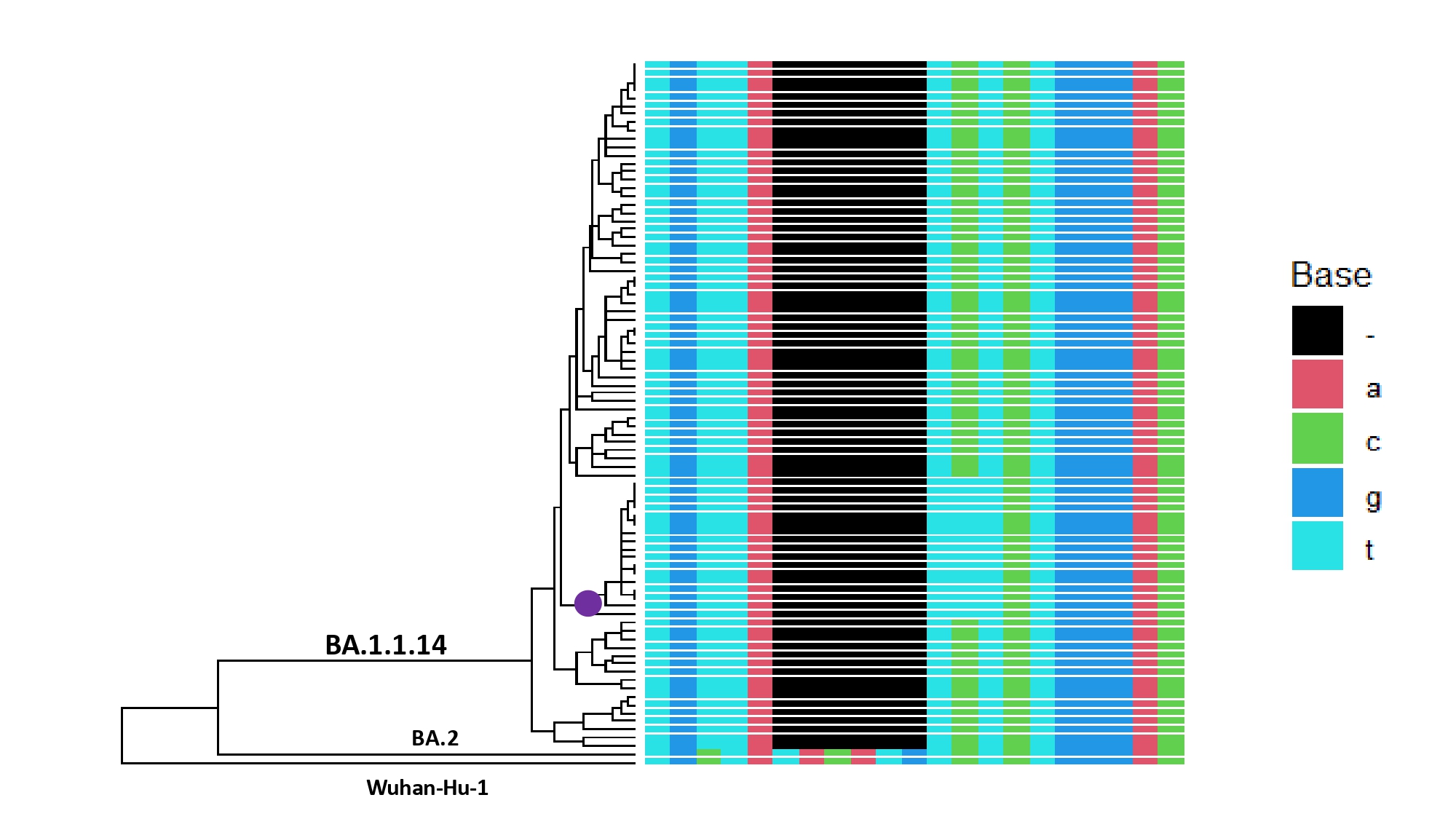


### **Supplementary data 1: Pipeline for bioinformatic analysis**

The reads were aligned to the reference MN908947.3 from Wuhan SARS-CoV-2 using BWA-mem. [(1)](https://www.zotero.org/google-docs/?rCOeKL) Trimming and identification of SNPs and indels (minimum quality threshold of 30, minimum read depth of 10) was performed with iVar. [(2)](https://www.zotero.org/google-docs/?i0ru45)

To build each consensus sequence, we merged the reads with SAMtools *mpileup* and then used iVar *consensus* with a minimum VAF threshold of 0.5. We assigned the consensus sequences to a SARS-CoV-2 clade and lineaje with Nextclade ([https://clades.nextstrain.org](https://clades.nextstrain.org/)) and Usher genome (https://genome.ucsc.edu). The global quality reports were generated using FastQC. [(3)](https://www.zotero.org/google-docs/?UgWvCd)

REFERENCES:

[1. Li H. Aligning sequence reads, clone sequences and assembly contigs with BWA-MEM [Internet]. arXiv; 2013 May [cited 2022 May 25]. Report No.: arXiv:1303.3997. Available from: http://arxiv.org/abs/1303.3997](https://www.zotero.org/google-docs/?D6Xufz)

[2. Grubaugh ND, Gangavarapu K, Quick J, Matteson NL, De Jesus JG, Main BJ, et al. An amplicon-based sequencing framework for accurately measuring intrahost virus diversity using PrimalSeq and iVar. Genome Biol. 2019 Jan 8;20(1):8.](https://www.zotero.org/google-docs/?D6Xufz)

[3. Andrews S. FastQC: a quality control tool for high throughput sequence data. 2010;](https://www.zotero.org/google-docs/?D6Xufz)
